# Calculation of the effect of tip geometry on noncontact atomic force microscopy using a qPlus sensor

**DOI:** 10.3762/bjnano.4.2

**Published:** 2013-01-08

**Authors:** Julian Stirling, Gordon A Shaw

**Affiliations:** 1 School of Physics and Astronomy, The University of Nottingham, University Park, Nottingham, NG7 2RD, United Kingdom; 2 Physical Measurement Laboratory, National Institute of Standards and Technology, Gaithersburg, Maryland 20899, USA

**Keywords:** atomic force microscopy, force spectroscopy, lateral forces, mechanical vibrations, qPlus

## Abstract

In qPlus atomic force microscopy the tip length can in principle approach the length of the cantilever. We present a detailed mathematical model of the effects this has on the dynamic properties of the qPlus sensor. The resulting, experimentally confirmed motion of the tip apex is shown to have a large lateral component, raising interesting questions for both calibration and force-spectroscopy measurements.

## Introduction

From imaging of individual chemical bonds [1] to subatomic resolution of the structure of the tip apex [2], many experiments have demonstrated the ability of qPlus atomic force microscopy (AFM) to produce unprecedented imaging resolution. Other qPlus studies have measured both the forces necessary to perform atomically precise manipulation [3–5], and the strength of both atomic and molecular interactions [6–7]. As with all forms of AFM, image resolution and force measurements ultimately depend on the structure of the last few angstroms of the tip apex [1–2,4,6–9]. The qPlus sensor is unusual for an AFM sensor in that it is constructed from a quartz tuning fork and has a tip comparable in length to the tine [10]. Hence, the macroscopic geometry of the tip also cannot be ignored [11].

The spring constant of the sensor must be known for any conversion from raw data to meaningful force measurements [12]. However, despite the quoted piconewton precision of qPlus measurements [3], the spring constant is often left unmeasured and is assumed to be *k* ≈ 1800 N·m^−1^ from the geometry of the bare tine [10]. Measurements of the spring constants of qPlus sensors have produced conflicting results [4,13], which highlights the need for more detailed analysis. Tung et al. [11] have shown that the dimensions of conical tips also have large effects on the dynamics of higher eigenmodes, suggesting that careful consideration of tip geometry is necessary for sensors operated in the second eigenmode or above.

In this paper we go further, providing a detailed analytical solution for the deflection, elastic potential energy, and spring constant of the tine of a qPlus sensor, for an arbitrary tip geometry. Furthermore, we derive the resulting lateral component of the motion of the tip apex, showing it, both theoretically and experimentally, to be comparable to the amplitude of motion normal to the surface even in the first eigenmode. This differs from previous work considering the lateral motion of qPlus sensors, such as Heyde et al. [14], as the lateral motion arises directly from the eigenmode used for imaging, rather than a parallel or torsional eigenmode. As such the lateral motion will not reveal itself by producing double images. The lateral motion has major implications for force measurements: whereas standard analysis assumes sensitivity to normal forces only [15], our analysis shows that both normal and lateral tip–sample interactions are sampled during qPlus imaging in the first eigenmode. We calculate the effect the lateral motion has on the measured frequency shift, and hence, how this affects calibration, imaging, and spectroscopy.

## Results and Discussion

Modelling the tine of the qPlus sensor as an Euler–Bernoulli beam [16] of length *L*, we can write

[1]
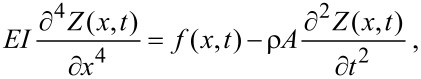


where *E*, *I*, ρ, and *A* are the Young’s modulus, second moment of area, density, and cross-sectional area of the tine, respectively. *f*(*x*,*t*) is the external force per unit length acting on the tine, and *Z*(*x*,*t*) is the deflection along the length of the tine. Separating the spatial (Φ*_i_*(*x*)) and temporal (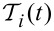) components of the deflection for all eigenmodes, *i*, we can write

[2]
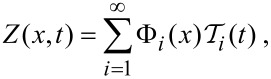


where

[3]
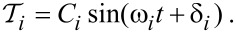


As Equation 1 is fourth-order spatially, we get the following general spatial solution:

[4]
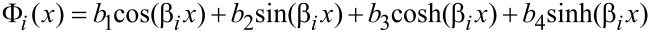


where β*_i_* is the spatial frequency of the *i*th mode governed by

[5]
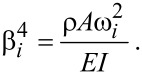


The tip connected to the tine, with mass *m*_tip_ and moment of inertia about the point of rotation of the tip 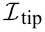 (A stylised 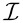 is used to differentiate between moments of inertia and moments of area *I*), will produce a resulting force of


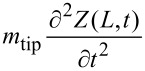


and torque of


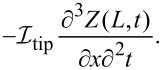


Thus, we can write the spatial boundary conditions as:

[6]
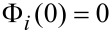


[7]
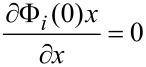


[8]
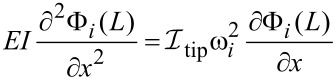


[9]
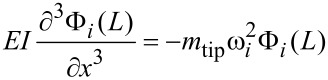


as the tine is fixed at *x* = 0. Under the assumption of uniform tip density we can write 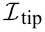 = *Jm*_tip_, where *J* is a constant with units of length squared. The very small effect of the motion, normal to the tine, of the center of mass of the tip due to the tip rotation has been ignored, as this significantly increases the complexity of the problem for a negligible component of the motion unless the width of the tip becomes significant compared to its entire length, including the portion attached to the end of the tine.

In Appendix A (see Supporting Information File 1), we apply these conditions to solve the spatial equation. From Equation 36 and Equation 37, the conditions *b*_1_ = −*b*_3_ and *b*_2_ = −*b*_4_ are apparent, and the ratio of *b*_1_ to *b*_2_ can be found from Equation 38. From this ratio we obtain the analytical form of the spatial solution as

[10]
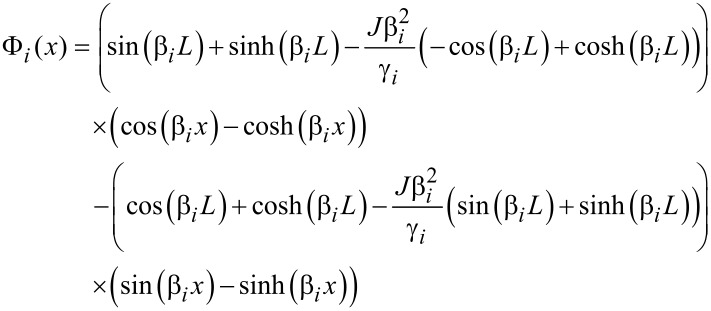


where γ*_i_* is defined in Equation 40.

### Effects on dynamic properties

As shown by Butt and Jaschke [17], properties of a cantilever (or tine) such as the dynamic spring constants and the proportion of energy in each eigenmode at thermal equilibrium can be found by considering the elastic potential energy of the tine. This becomes particularly important if calibration is done by thermal tuning, or if force measurements are performed by using higher modes [11]. The elastic potential energy of the tine is given by

[11]
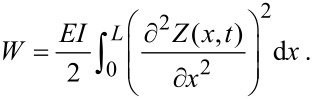


Again, separating into spatial and temporal components gives

[12]
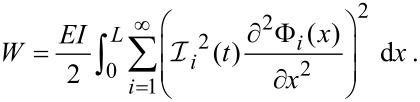


In Appendix B (see Supporting Information File 1), we show that, just as for bare cantilevers [17], this reduces to

[13]
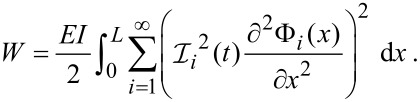


For brevity we define 
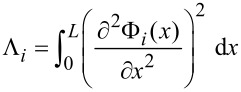
.

This allows us to write the average elastic potential energy for each eigenmode as

[14]
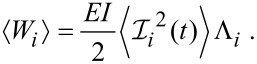


In thermal equilibrium, the equipartition theorem requires that 
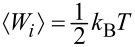
. But, as 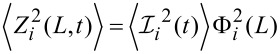, and as we include the static spring constant from Euler–Bernoulli beam theory, *k*_stat_ = 3*EI*/*L*^3^, we obtain the mean square deflection of each mode in thermal equilibrium as

[15]
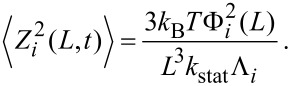


The full analytical form of 
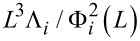
 is derived in Appendix C (see Supporting Information File 1). Combined with values for β*_i_* (solutions of Equation 42 in Appendix A, see Supporting Information File 1), this could be used to measure the static spring constant of a qPlus sensor with a well-defined tip geometry by thermal tuning. However, thermal tuning of qPlus sensors with spring constants on the order of 2 kN·m^−1^ remains a challenging experimental task as the rms amplitude of thermal excitation at 300 K is ≈1.4 pm.

For well-calibrated force measurements, the dynamic spring constant for the excited eigenmode *k_i_* must be calculated. By considering the equipartition theorem again, but with the dynamic spring constant and Hooke’s law, 
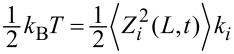
. Combining with Equation 15 this gives

[16]
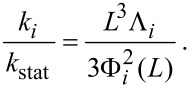


Thus, from the results in Appendix C (see Supporting Information File 1), as with the thermal tuning, the dynamic spring constant for any eigenmode can be calculated, provided the tip geometry is well defined, and the static spring constant is known.

**Figure 1:**
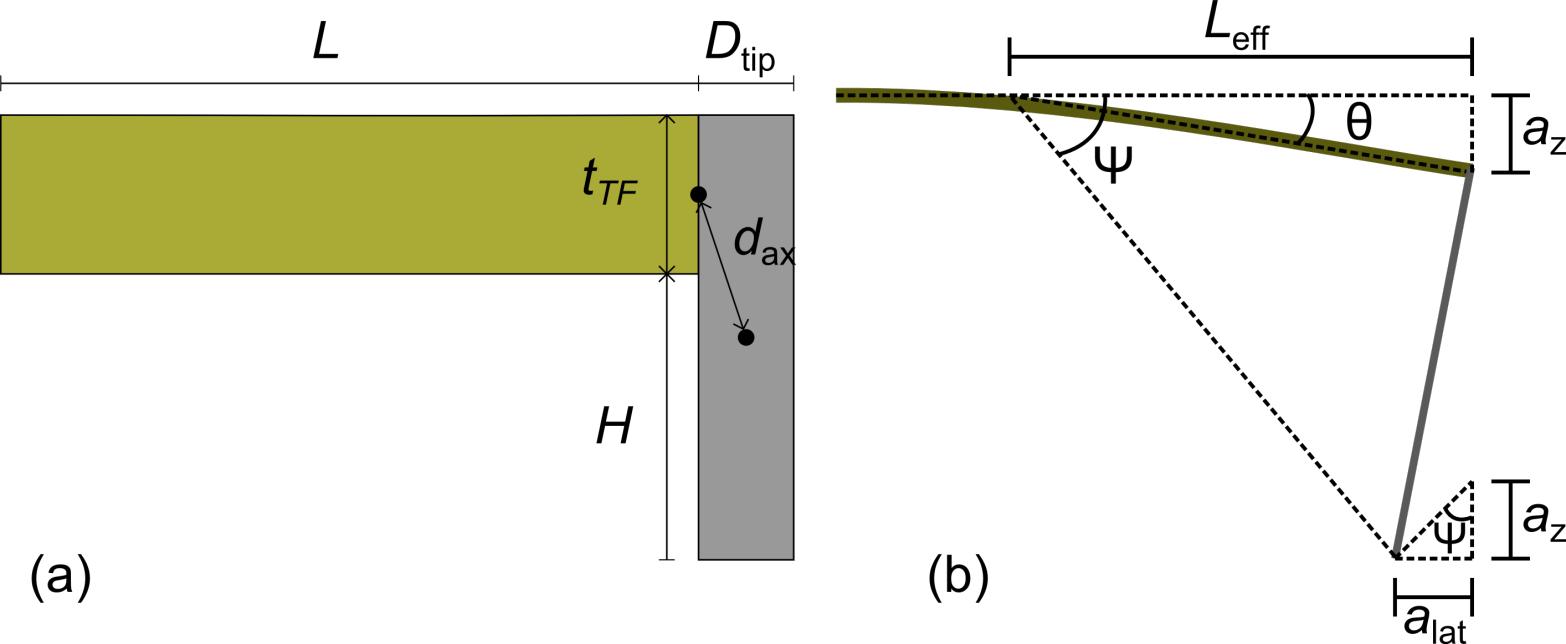
(a) Diagram of theoretical model. *d*_ax_ is the distance from the centre of mass of the tip to the axis of tip rotation. The tine of the tuning fork is assumed to be clamped at the left-hand side. (b) Geometrical diagram of the tip motion as the cantilever deforms. Note the two ψ angles are not identical in this diagram as θ is too large due to the exaggerated amplitude of deflection, *a_z_*.

The tine of a qPlus sensor was modeled as a quartz beam of length *L* = 2.4 mm, width *w* = 130 μm, and thickness *t*_TF_ = 214 µm (Figure 1a). The tip was modeled as a tungsten cylinder of diameter *D*_tip_ attached to the end of the tine of the tuning fork. The dynamics of the tip bending have not been included as the model is only of interest at the eigenfrequencies of the loaded tine. The tip protrudes *H* from the tine giving the tip a total length of *H* + *t*_TF_. The axis of rotation is located at the center of the join between the tip and cantilever. By the parallel-axis theorem, the moment of inertia, 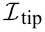, can be calculated as 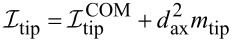, where 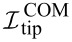 is the moment of inertia through the center of mass of the tip and *d*_ax_ is the distance from the axis of rotation to the center of mass. The distance to the axis is 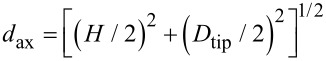. Thus, the moment of inertia about the axis of rotation is

[17]
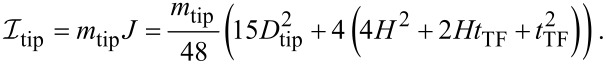


**Figure 2:**
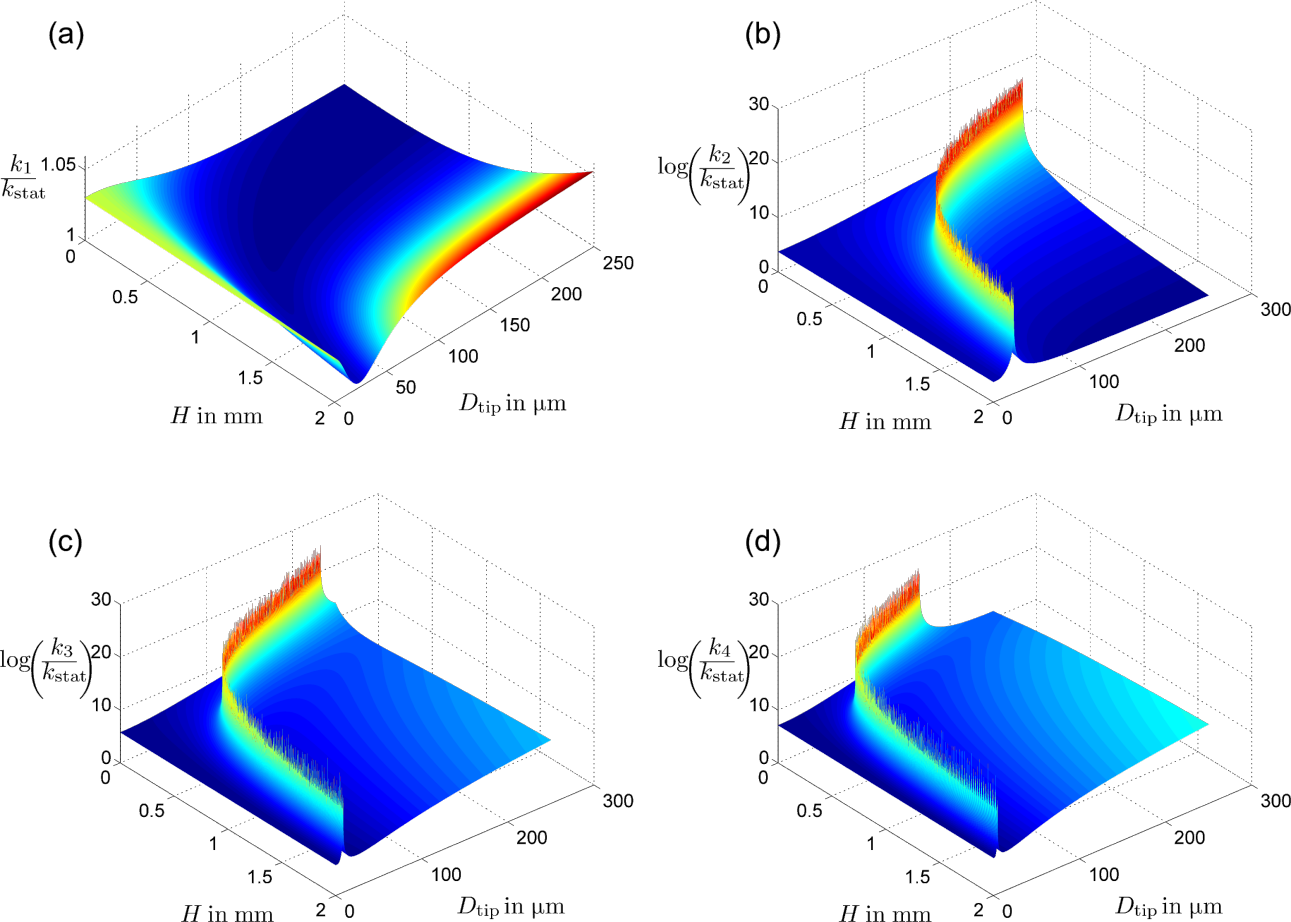
The ratio of dynamic spring constants *k_n_* to the cantilever static spring constant *k*_stat_ for *n* = 1,2,3,4, plotted for a range of tip lengths and diameters. The sudden increases in the higher eigenmodes result from nodes positioned at the end of the tip resulting in infinite spring constants.

Using this model, dynamic spring constants were calculated for the first four eigenmodes, relative to *k*_stat_ (Figure 2). The first two eigenmodes agree qualitatively with experimentally verified Hamiltonian calculations by Tung et al. for the first two eigenmodes of conical tips [11]. The sudden rises to infinite spring constant correspond to when a node of the vibrational mode is located at the end of the cantilever.

### Resulting lateral motion

The model presented above allows us to calculate the angle the tip rotates through during the oscillation by using

[18]
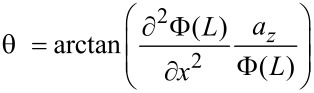


[19]
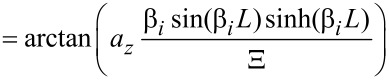


where *a_z_* is the amplitude of oscillation, and Ξ is a dimensionless parameter defined as

[20]
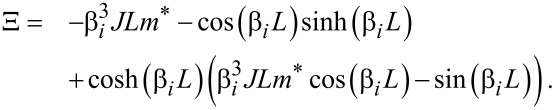


m^*^ is the ratio of tip mass to the mass of the tine.

**Figure 3:**
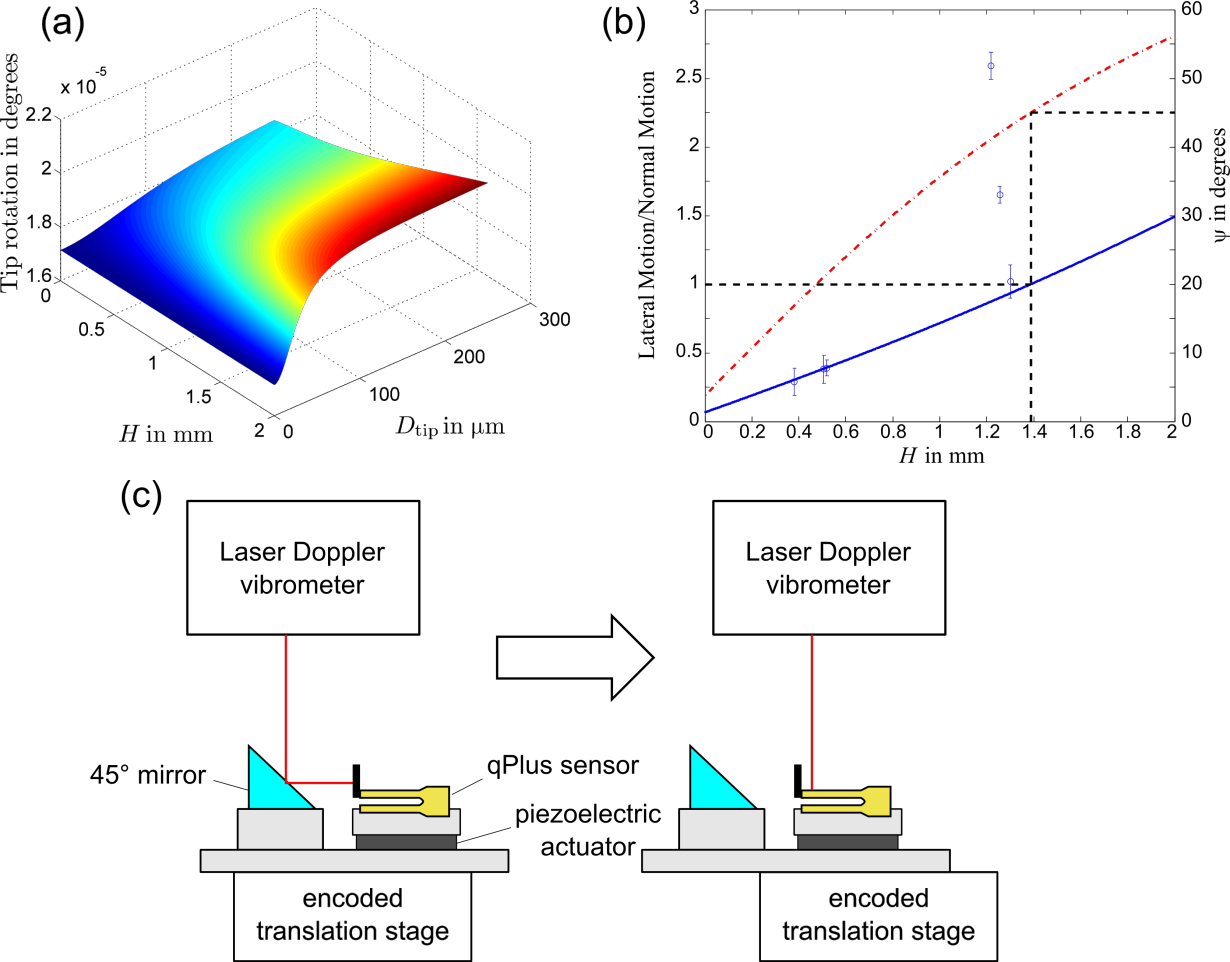
Effect of tip geometry on tip movement for an arbitrary oscillation amplitude. (a) Angle of tip rotation plotted for varying tip sizes. (b) Amplitude of lateral motion of the tip apex plotted (solid line) for varying lengths with a tip diameter of 50 μm. Circular data points represent experimentally measured values. Angle of resulting motion, ψ of the tip apex is also plotted (dot-dashed line). The dashed lines represent where the lateral amplitude equals the normal amplitude. Error bars represent 1 standard error. (c) Schematic of the experimental setup for measuring both normal and lateral motion of the qPlus sensor.

This has been plotted in Figure 3a for the first eigenmode with an amplitude of 0.5 nm. The angle is extremely small, about (2 × 10^−5^)°, and nearly constant for different tip geometries. However, the lateral motion at the end of the tip will have an amplitude of

[21]
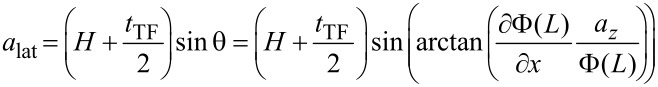


[22]
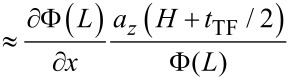


[23]
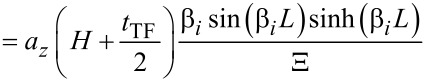


The calculated amplitude of lateral vibrations has been plotted as a function of *H* in Figure 3b, for *D*_tip_ = 50 μm, as the angular dependence on the tip diameter is relatively small. It is apparent that this lateral motion can be significant compared with the normal motion, even for relatively short tips, reaching an equal amplitude at *H* = 1.389 mm. We can define *a*, the total amplitude of oscillation at the apex, as

[24]
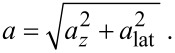


Defining the angle of tip motion as ψ, where ψ = 0 corresponds to oscillation normal to the surface and ψ = 90° to oscillation parallel to the surface, we obtain

[25]
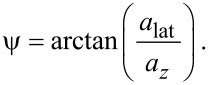


It is important to note that as the angle of tip rotation is so small, the motion of the tip apex should not be thought of as circular motion, but instead the tip apex is moving over a linear trajectory at an angle ψ to the surface, with negligible rotation.

#### Experimental validation

The resulting lateral motion was measured experimentally for Omicron Nanotechnology qPlus sensors excited mechanically from below by a piezoelectric actuator (Figure 3c). The actuator was driven by a digital lock-in amplifier (Perkin Elmer 7280 DSP). The deflection of the qPlus sensor was measured by using a laser Doppler vibrometer (Polytec OFV-522) connected to a lock-in amplifier. The qPlus sensor and piezoelectric actuator was mounted on an encoded translation stage allowing deflection measurements to be recorded in multiple positions on the sensor. A 45° mirror positioned near the qPlus sensor allowed lateral deflection measurements of the tip to be made without remounting the sensor or interrupting the excitation, thus limiting changes to the transfer function.

Tungsten wire of 50 μm in diameter was attached to the end of the bare qPlus sensor with EPO-TEK H21D electrically conductive silver epoxy. The tip wire was then etched to different lengths by using a Gamry potentiostat operating as a chronocoulometer with a platinum mesh counter electrode and a saturated calomel reference electrode (SCE). The integrated etch current allowed some degree of control over the quantity of material removed from the tip [18]. The sensor was positioned with a micromanipulator such that the 1 M KOH electrolyte contacted only the tungsten probe tip, then a potential of 0.3 V versus SCE was applied until the desired charge was accumulated from the Faradaic etch current, and the sensor was then removed from the solution. A subsequent five-minute rinse in deionized water was performed in a similar fashion, without application of an external potential.

For measurements of both normal and lateral motion the translation stage was used to collect multiple deflection readings at different positions along the sensor and tip. These were then extrapolated to get the deflection at the end of the sensor and tip despite poor reflectivity at both regions of the sensor not covered by gold electrodes and at the tip apex where it was etched.

The ratio of lateral to normal motion was measured for six sensors. Tip lengths were measured with an optical microscope and an encoded translation stage. These results are presented in Figure 3b. Four of the six sensors show good agreement with the theoretical curve. The two remaining sensors show significantly higher lateral motion. This is most likely due to compliance in the epoxy used to connect the tip, allowing some rotation of the tip relative to the end of the sensor. These results confirm the prediction of large lateral motions on qPlus sensors.

Lateral amplitudes of this magnitude, at first glance, could be thought to limit the resolution of the AFM. However, it is important to consider that the lateral motion is perfectly correlated to the normal motion, unlike lateral motion resulting from torsional modes of the cantilever, which will oscillate with much higher frequency causing blurring of the image. Simulated images and spectra can be generated to theoretically calculate the effect; however, one must carefully consider both the amplitude calibration and the methods for calculating frequency shifts from a potential before continuing.

#### Effect on frequency shift

Under the assumption that the direction of motion of the tip apex is parallel to the motion of the end of the cantilever (or tine) it can be shown [9] that

[26]
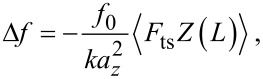


where *Z*(*L*) = *a_z_*cos(ω_0_*t*), and *F*_ts_ is the force due to the tip–sample interaction.

In the case that the motion of the tip is not parallel to the oscillation of the cantilever, more care must be taken. Equation 26 can be derived from Newton’s second law in the reference frame of the end of the cantilever

[27]
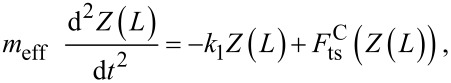


where 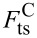 is the force due to the tip–sample interaction as experienced at the end of the cantilever, and *m*_eff_ is the effective mass of the tip and cantilever. Thus, if Equation 26 is modified to include lateral oscillations, the amplitude terms will remain as *a_z_*, as this is the oscillation amplitude of the beam. However, the tip–sample force must be modified from the interaction at the tip apex to the resulting force at the end of the cantilever.

For an amplitude of *a_z_*, the end of the cantilever is angled by θ at its maximum deflection, as shown in Figure 1b, which can be treated as circular motion about an effective pivot at a distance of *L*_eff_ = *a_z_*/tan θ from the end of the cantilever. Combining with Equation 21 and Equation 25, and equating sin θ to tan θ due to the very small angle we can show that

[28]
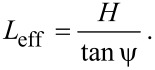


Thus, the bending can be described by a torque of

[29]
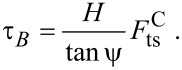


In the reference frame of the tip apex the distance to the effective pivot is *H*/sin ψ by simple geometry, and the measured tip–sample force *F*_ts_ is perpendicular to the vector from the tip apex to the effective pivot, such that

[30]
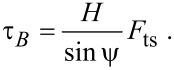


Hence,

[31]
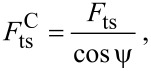


and so the frequency shift can be calculated as

[32]
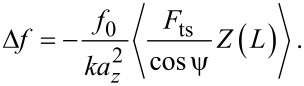


In the case that the lateral force is zero,

[33]
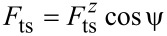


where 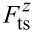 is the *z* component of the tip–sample force. Thus, if the calibrated amplitude of the oscillation is *a_z_*, rather than the total amplitude of oscillation at the tip apex *a*, then Δ*f* is equal to the expected result for tip motion parallel to the cantilever oscillation. However, if lateral forces are present, then these will also affect the frequency shift.

#### Effect on calibration

Amplitude calibration in qPlus AFM is usually performed by measuring the *z* extension needed to maintain a constant value for 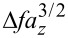 [10]. As it can be shown for large amplitudes, by inserting

[34]
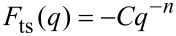


into Equation 32, where *q* is the position of the tip apex relative to the surface, that

[35]
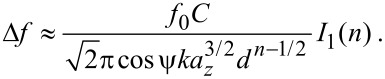


This follows from using the force conversion in Equation 31, and applying the method from [15]. *I*_1_(*n*) is an integral, dependent on *n*, but constant for the experiment, and *d* is the point of closest approach.

To maintain a constant 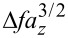, the point of closest approach must be kept constant. The recorded *z* extension to meet this condition will be equal to the change in *a_z_*. Thus, the calibrated amplitude is not the amplitude of the complete motion of the tip apex, but rather *a_z_*, the *z* component of this amplitude.

#### Effect on imaging and spectroscopy

**Figure 4:**
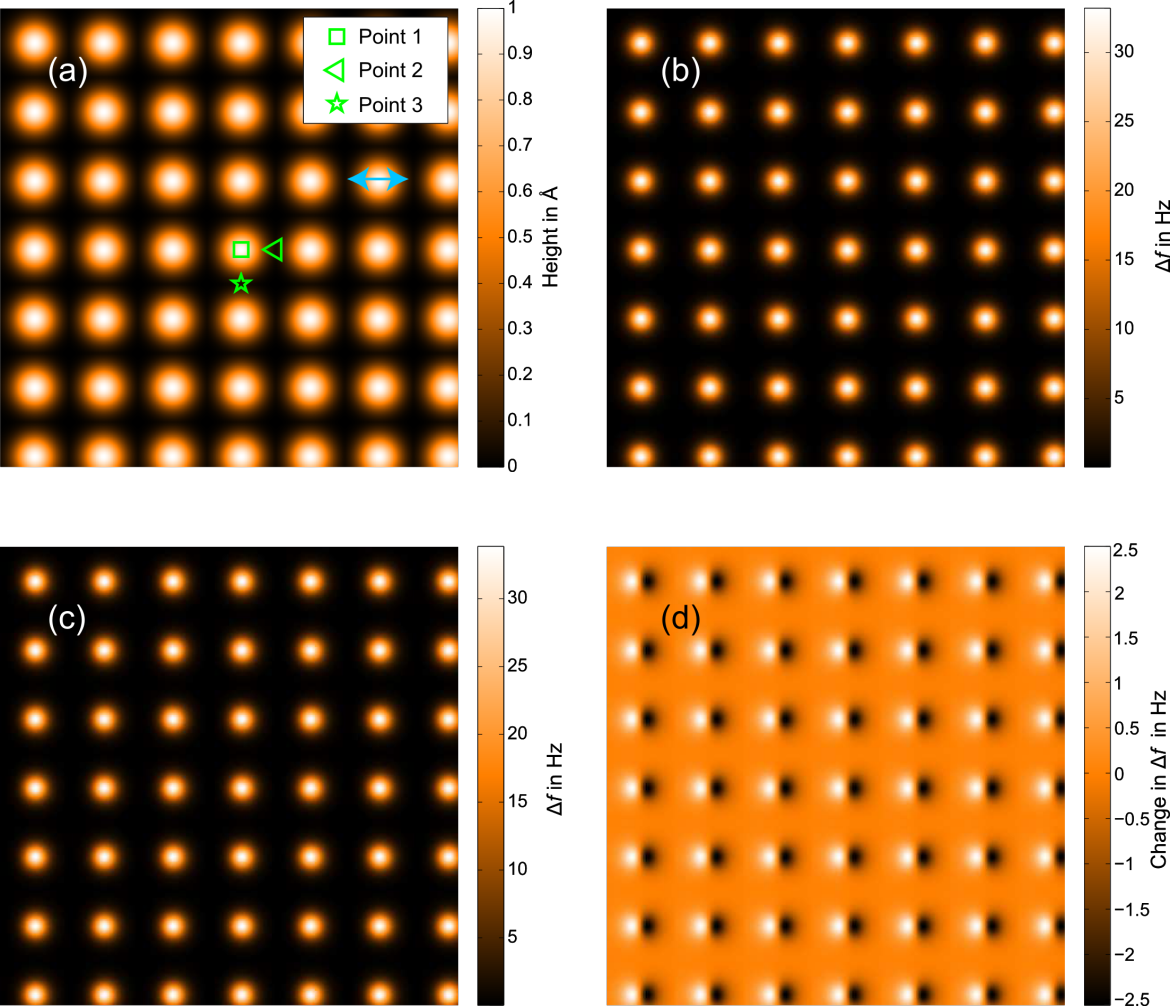
Effect of tip motion on imaging for an oscillation amplitude of *a_z_* = 5 Å. All image widths are 2 nm × 2 nm. (a) shows the model surface. (b) and (c) show theoretical Δ*f* images for constant height scans with tip motion normal and at 45° to the sample surface, respectively. Lateral motion of the tip in (c) is aligned with the *x* axis of the scan, as indicated by the light blue arrow in (a). (d) is the difference between scans (b) and (c).

Simulated AFM data were produced by creating a Lennard-Jones potential for a simple 2-D square lattice, with a lattice constant of 3 Å, and a minimum potential of −3 eV at a distance of 0.5 Å (Figure 4a). For simplicity the simulated AFM was run in constant height mode to collect Δ*f* images. These were calculated by using Equation 32. Images were collected for both tips oscillating normal and at 45° to the sample (i.e., a qPlus sensor with a tip length of 1.389 mm). An oscillation amplitude of *a_z_* = 0.5 Å was used for both motions (Figure 4b and Figure 4c), thus mimicking a calibration performed using the method described in the previous section. This leads to a total amplitude of *a* = 0.707 Å for the angled motion of the finite tip. Theses scans were aligned such that the position of closest approach of the tip apex is the same for both scans.

Qualitatively the images for both angled and normal tip motion look almost identical, and there is no noticeable reduction in resolution. Quantitatively the difference between the scans (Figure 4d) is ±2.5 Hz for images with a Δ*f* range of approximately 34 Hz, giving rise to a relative error of less than 8%.

As the model potential has only one decay power rather than a combination of long-range and short-range forces, the relative changes between angled and normal oscillations are largely independent of the amplitude of oscillation. The change in sensitivity that arises from the different amplitudes will affect the absolute values of the frequency shift; however, as the simulation is not subject to limits in frequency resolution from experimental noise, these absolute values are of little interest.

**Figure 5:**
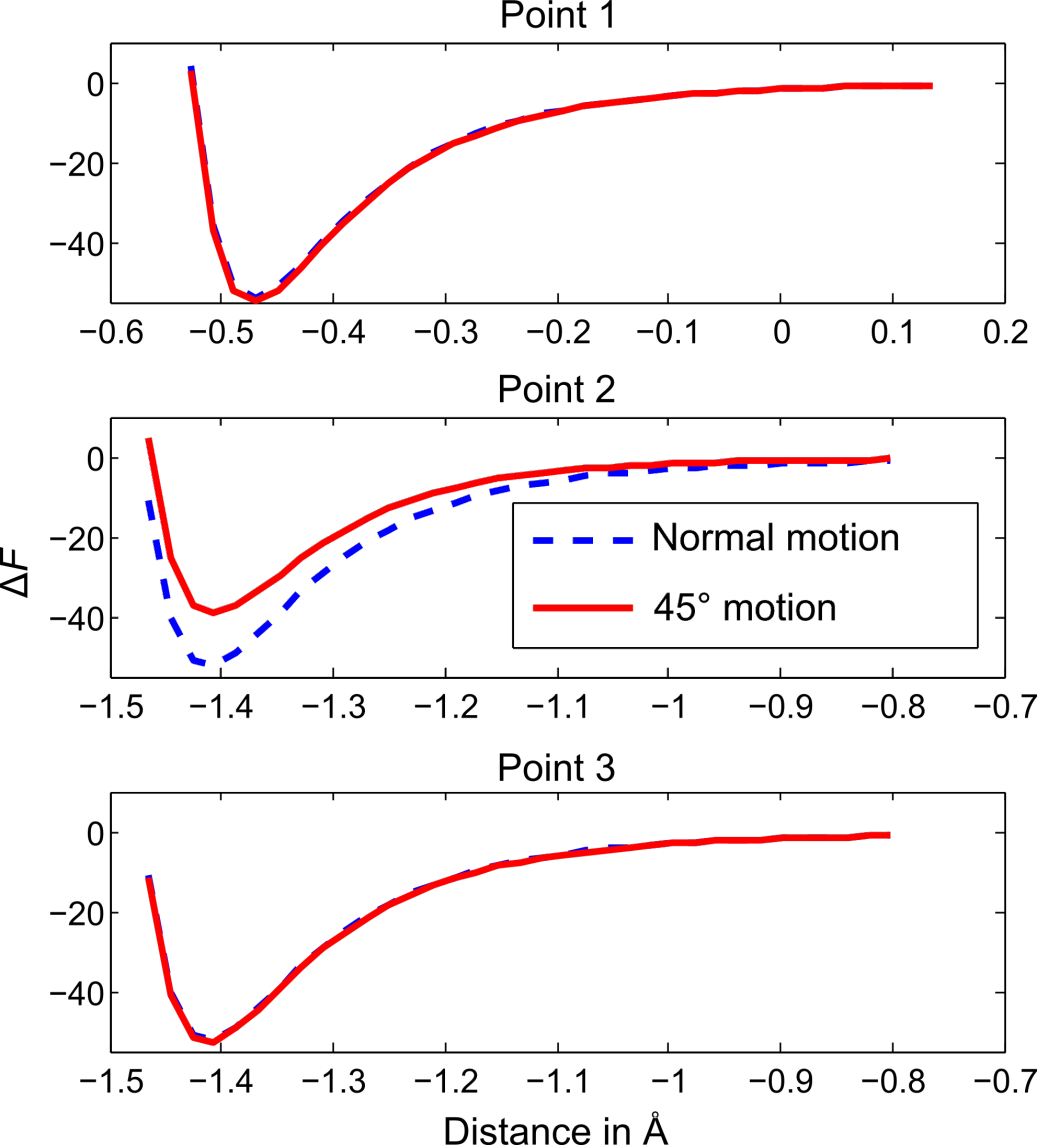
Effect tip motion on spectroscopy for an oscillation amplitude of *a_z_* = 5 Å. Simulated spectroscopy measurements taken at three points as marked on Figure 4a. These show that only when the tip apex moves through significant lateral forces does the angle of scan have a noticeable effect on the *z*-spectroscopy.

Following this, simulated *z*-spectroscopy measurements were taken over three points (marked on Figure 4a) for both images. The results presented in Figure 5 show that for points 1 and 3, where the lateral force is near zero throughout the tip oscillation, the spectra align with a relative error of less than 3% at the point of highest interaction. However, for point 2 where the lateral force is significant as the tip moves diagonally over the adjacent atom, there is a difference of 23% between the curves at the point of highest interaction.

## Conclusion

We have shown that using a simple Euler–Bernoulli model for the tine of a qPlus sensor, and inserting boundary conditions that account for both the moment of inertia and mass of the tip, we were able derive analytical results for a range of dynamic sensor properties. When the moment of inertia of the tip is zero our results agree with previous results found in the literature for point-mass weighted cantilevers [19]. After including the moment of inertia our results are in agreement with Tung et al. [11].

Further analysis of our model revealed large lateral motion at the tip apex when the tip length is comparable to the cantilever length. Our model was validated by direct experimental measurement of the lateral tip motion of a resonant qPlus sensor. To maintain a ratio of lateral to normal motion of under 10% the tip must protrude less that 57 μm from the tine for qPlus sensors of the modelled geometry. The methods currently used to calibrate amplitude in these systems accurately describe the *z*-component of amplitude; however, the overall amplitude of motion at the tip apex is larger. If the lateral force is near zero throughout the tip oscillation then this has minimal effect on either imaging or spectroscopy measurements, thus explaining the ability of qPlus AFM to gain both subangstrom spatial resolution, and agreement with theoretical force measurements [7]. However, the lateral motion has a large effect on any data when lateral forces are present, requiring both careful analysis of experimental results and knowledge of the sensor and tip geometry.

## Supporting Information

File 1 Further details of the presented theoretical model
