# Supplementary material for: Calculation of the effect of tip geometry on noncontact atomic force microscopy using a qPlus sensor
Source: Beilstein J Nanotechnol. 2013 Jan 8;4:10–9. doi: 10.3762/bjnano.4.2 (PMC3566854; doi:10.3762/bjnano.4.2)
Supplement: File 1 — Further details of the presented theoretical model [file Beilstein_J_Nanotechnol-04-10-s001.pdf]

Supporting Information

for

Calculation of the effect of tip geometry on noncontact atomic force microscopy using a qPlus sensor

Julian Stirling*[1] and Gordon A. Shaw[2]

Address: [1]School of Physics and Astronomy, The University of Nottingham, University Park, Nottingham, NG7 2RD, United Kingdom and [2]Physical Measurement Laboratory, National Institute of Standards and Technology, Gaithersburg, Maryland 20899

Email: Julian Stirling* - ppxjs1@nottingham.ac.uk

* Corresponding author

Further details of the presented theoretical model

Appendix A

The boundary conditions in Equations 6–9 allow us to form four simultaneous equations for the factors $b_{1..4}$ of $\Phi_i(x)$ from Equation 4.

$$b_1 + b_3 = 0 \tag{36}$$

$$b_2 + b_4 = 0 \tag{37}$$

$$\begin{aligned} b_1 \left(-\cos(\beta_i L) + \frac{J\beta_i^2}{\gamma_i} \sin(\beta_i L) \right) + b_2 \left(-\sin(\beta_i L) - \frac{J\beta_i^2}{\gamma_i} \cos(\beta_i L) \right) \\ + b_3 \left(\cosh(\beta_i L) - \frac{J\beta_i^2}{\gamma_i} \sinh(\beta_i L) \right) + b_4 \left(\sinh(\beta_i L) - \frac{J\beta_i^2}{\gamma_i} \cosh(\beta_i L) \right) = 0 \end{aligned} \tag{38}$$

$$\begin{aligned} b_1 \big(\gamma_i \sin(\beta_i L) + \cos(\beta_i L) \big) + b_2 \big(-\gamma_i \cos(\beta_i L) + \sin(\beta_i L) \big) \\ + b_3 \big(\gamma_i \sinh(\beta_i L) + \cosh(\beta_i L) \big) + b_4 \big(\gamma_i \cosh(\beta_i L) + \sinh(\beta_i L) \big) = 0 \end{aligned} \tag{39}$$

where γ_i is a dimensionless parameter defined as

$$\gamma_i = \frac{EI\beta^3}{m_{\text{tip}}\omega_i^2}. \tag{40}$$

Writing Equations 36, 37, 38, and 39 as a matrix equation in the form

$$\underline{\underline{D}} \begin{pmatrix} b_1 \\ b_2 \\ b_3 \\ b_4 \end{pmatrix} = \begin{pmatrix} 0 \\ 0 \\ 0 \\ 0 \end{pmatrix} \tag{41}$$

it becomes clear that if $\det(\underline{\underline{D}}) \neq 0$, then the solution would trivially be $b_{1..4} = 0$, a stationary beam. Hence, $\det(\underline{\underline{D}}) = 0$, giving the following condition for β_i:

$$\begin{aligned} 1 + m^{*2}\beta_i^4 JL^2 - \left(1 + m^{*2}\beta_i^4 JL^2\right) \cos(\beta L) \cosh(\beta_i L) \\ + m^* \beta_i L \left[\left(1 - J\beta_i^2\right) \cos(\beta_i L) \sinh(\beta_i L) - (1 + J\beta_i) \cosh(\beta_i L) \sin(\beta_i L) \right] = 0 \end{aligned} \tag{42}$$

using

$$\gamma_i = \frac{\rho A}{\beta_i m_{\mathrm{tip}}} = \frac{1}{\beta_i L m^*}, \tag{43}$$

from Equations 5 and 40, where m^* is the ratio of the tip mass to the mass of the tine. Equation 42 can be numerically solved simply and quickly by using the Newton-Raphson method.

Appendix B

Equation 12 can be expanded to give

$$W = \frac{EI}{2}\left[\sum_{i=1}^{\infty}\mathscr{T}_i^2(t)\int_0^L\left(\frac{\mathrm{d}^2\Phi_i(x)}{\mathrm{d}x^2}\right)^2\mathrm{d}x + \sum_{i=1}^{\infty}\sum_{k=1,k\neq i}^{\infty}\mathscr{T}_i(t)\mathscr{T}_k(t)\int_0^L\frac{\mathrm{d}^2\Phi_i(x)}{\mathrm{d}x^2}\frac{\mathrm{d}^2\Phi_k(x)}{\mathrm{d}x^2}\mathrm{d}x\right] \tag{44}$$

Butt and Jaschke [1] use

$$\frac{\mathrm{d}^4\Phi_i(x)}{\mathrm{d}x^4} = \beta_i^4\Phi_i(x) \tag{45}$$

(which is clearly still true for our boundary conditions from the general form of Φ given in Equation 4) to show that the integral with mixed ($\Phi_i(x)$ and $\Phi_k(x)$) second derivatives can be written as

$$\int_0^L\frac{\mathrm{d}^2\Phi_i(x)}{\mathrm{d}x^2}\frac{\mathrm{d}^2\Phi_k(x)}{\mathrm{d}x^2}\mathrm{d}x = \frac{1}{\beta_i^4-\beta_k^4}\left[\beta_i^4\frac{\mathrm{d}^2\Phi_k(x)}{\mathrm{d}x^2}\frac{\mathrm{d}\Phi_i(x)}{\mathrm{d}x} - \beta_i^4\frac{\mathrm{d}^3\Phi_k(x)}{\mathrm{d}x^3}\Phi_i(x) - \beta_k^4\frac{\mathrm{d}^2\Phi_i(x)}{\mathrm{d}x^2}\frac{\mathrm{d}\Phi_k(x)}{\mathrm{d}x} + \beta_k^4\frac{\mathrm{d}^3\Phi_i(x)}{\mathrm{d}x^3}\Phi_k(x)\right]_0^L. \tag{46}$$

If we combine Equations 40 and 43 to give

$$\beta_i^4 L m^* = \frac{m_{\text{tip}} \omega_i^2}{EI} \tag{47}$$

then we can rewrite boundary conditions Equations 8 and 9 as

$$\frac{\partial^2 \Phi_i(L)}{\partial x^2} = \beta_i^4 J L m^* \frac{\partial \Phi_i(L)}{\partial x} \tag{48}$$

$$\frac{\partial^3 \Phi_i(L)}{\partial x^3} = -\beta_i^4 L m^* \Phi_i(L)\,. \tag{49}$$

From these conditions it becomes clear that the first and third terms in the square brackets of Equation 46 cancel, as do the second and fourth. Thus,

$$\int_0^L \frac{\mathrm{d}^2 \Phi_i(x)}{\mathrm{d}x^2} \frac{\mathrm{d}^2 \Phi_k(x)}{\mathrm{d}x^2} \mathrm{d}x = 0\,, \tag{50}$$

and

$$W = \frac{EI}{2} \sum_{i=1}^{\infty} \mathscr{T}_i^2(t) \int_0^L \left(\frac{\partial^2 \Phi_i(x)}{\partial x^2} \right)^2 \mathrm{d}x\,. \tag{51}$$

Appendix C

To solve Λ_i we first integrate by parts twice to give

$$\Lambda_i = \left[\frac{\mathrm{d}\Phi_i(x)}{\mathrm{d}x} \frac{\mathrm{d}^2 \Phi_i}{\mathrm{d}x^2} - \Phi_i(x) \frac{\mathrm{d}^3 \Phi_i(x)}{\mathrm{d}x^3} \right]_0^L + \int_0^L \Phi_i(x) \frac{\mathrm{d}^4 \Phi_i(x)}{\mathrm{d}x^4} \mathrm{d}x\,. \tag{52}$$

The square brackets can be evaluated using boundary conditions from Equations 6, 7, 48, and 49. Furthermore, with Equation 45 the integral can be written in terms of $\Phi_i(x)$ only:

$$\Lambda_i = \beta_i^4 L J m^* \left(\frac{\mathrm{d}\Phi_i(L)}{\mathrm{d}x} \right)^2 + \beta_i^4 L m^* \Phi_i^2(L) + \beta_i^4 \int_0^L \Phi_i(x)^2 \mathrm{d}x\,. \tag{53}$$

The integral can be solved by substituting in $\zeta = x/L$, and writing $\beta_i L$ as α_i,

$$
\begin{aligned}
\int_0^L \Phi_i^2 \mathrm{d}x =& L\int_0^1 \Bigg\{ \left[\sin(\alpha_i) + \sinh(\alpha_i) - \frac{J\beta_i^2}{\gamma_i}\Big(-\cos(\alpha_i) + \cosh(\alpha_i) \Big) \right] \\
& \times \big(\cos(\alpha_i\zeta) - \cosh(\alpha_i\zeta) \big) \\
& - \left[\cos(\alpha_i) + \cosh(\alpha_i) - \frac{J\beta_i^2}{\gamma_i}\Big(\sin(\alpha_i) + \sinh(\alpha_i) \Big) \right] \\
& \times \big(\sin(\alpha_i\zeta) - \sinh(\alpha_i\zeta) \big) \Bigg\}^2 \mathrm{d}\zeta \qquad (54) \\
=& L\Big(\sin(\alpha_i) + \sinh(\alpha_i) \Big)^2 \\
& + \frac{3L}{\alpha_i}\Big(1 + \cos(\alpha_i)\cosh(\alpha_i) \Big) \\
& \times \Big(-\cosh(\alpha_i)\sin(\alpha_i) + \cos(\alpha_i)\sinh(\alpha_i) \Big) \\
& - \frac{J\beta_i^2}{\gamma_i} L \Bigg\{ \frac{1}{\alpha_i}\Big[-4 + \cos(2\alpha_i) + \Big(1 + 2\cos(2\alpha_i) \Big)\cosh(2\alpha_i) \Big] \\
& - 2(\cos(\alpha_i) - \cosh(\alpha_i))(\sin(\alpha_i) + \sinh(\alpha_i)) \Bigg\} \\
& + \frac{J^2\beta_i^4}{\gamma_i^2} L \Bigg[\Big(\cos(\alpha_i) - \cosh(\alpha_i) \Big)^2 \\
& + \frac{3}{\alpha_i}\Big(-1 + \cos(\alpha_i)\cosh(\alpha_i) \Big) \\
& \times \Big(\cosh(\alpha_i)\sin(\alpha_i) + \cos(\alpha_i)\sinh(\alpha_i) \Big) \Bigg]. \qquad (55)
\end{aligned}
$$

Returning back to notation without α_i or γ_i and using Equation 42 to replace $(1 + \cos(\beta_i L)\cosh(\beta_i L))$ in the second term, after some manipulation gives

$$
\begin{aligned}
=&L\Big(\sin(\beta_i L)+\sinh(\beta_i L)\Big)^2\\
&-3Lm^*\Big(\cosh(\beta_i L)\sin(\beta_i L)-\cos(\beta_i L)\sinh(\beta_i L)\Big)^2\\
&-\beta_i^2 Jm^* L\Bigg[-\frac{5}{2}+\cos(2\beta_i L)+\cosh(2\beta_i L)\\
&+\frac{1}{2}\cos(2\beta_i L)\cosh(2\beta_i L)\\
&-2\beta_i L\Big(\cos(\beta_i L)-\cosh(\beta_i L)\Big)\Big(\sin(\beta_i L)+\sinh(\beta_i L)\Big)\\
&-3m^*\beta_i L\Big(1-\cos(\beta_i L)\cosh(\beta_i L)\Big)\\
&\times\Big(\cosh(\beta_i L)\sin(\beta_i L)-\cos(\beta_i L)\sinh(\beta_i L)\Big)\Bigg]\\
&+\beta_i^6 J^2 m^{*2} L^3\Bigg[\Big(\cos(\beta_i L)-\cosh(\beta_i L)\Big)^2\Bigg]\\
&+3\beta_i^5 J^2 m^{*2} L^2\Bigg[\Big(-1+\cos(\beta_i L)\cosh(\beta_i L)\Big)\\
&\times\Big(\cosh(\beta_i L)\sin(\beta_i L)+\cos(\beta_i L)\sinh(\beta_i L)\Big)\Bigg].
\end{aligned}
\tag{56}
$$

Using Equation 42 again, this time to replace $\beta_i^3 Jm^* L(\cosh(\beta_i L)\sin(\beta_i L)+\cos(\beta_i L)\sinh(\beta_i L))$ in the final term, and rearranging will give

$$\begin{aligned}\int_0^L \Phi_i^2 \mathrm{d}x =& L\Big(\sin(\beta_i L)+\sinh(\beta_i L)\Big)^2\\ & -3Lm^*\Big(\cosh(\beta_i L)\sin(\beta_i L)-\cos(\beta_i L)\sinh(\beta_i L)\Big)^2\\ & -\beta_i^2 Jm^* L\Big[\sin^2(\beta_i L)\sinh^2(\beta_i L)\\ & -2\beta_i L\Big(\cos(\beta_i L)-\cosh(\beta_i L)\Big)\times\Big(\sin(\beta_i L)+\sinh(\beta_i L)\Big)\\ & -6m^*\beta_i L\Big(1-\cos(\beta_i L)\cosh(\beta_i L)\Big)\\ & \times\Big(\cosh(\beta_i L)\sin(\beta_i L)-\cos(\beta_i L)\sinh(\beta_i L)\Big)\Big]\\ & +\beta_i^6 J^2 m^{*2} L^3\Bigg[\Big(\cos(\beta_i L)-\cosh(\beta_i L)\Big)^2\\ & -3m^*\Big(1-\cos(\beta_i L)\cosh(\beta_i L)\Big)^2\Bigg]\,.\end{aligned} \tag{57}$$

It can be shown simply that

$$\left(\frac{\mathrm{d}\Phi_i(L)}{\mathrm{d}x}\right)^2 = 4\beta_i^2\sin^2(\beta_i L)\sinh^2(\beta_i L)\,, \tag{58}$$

and that

$$\begin{aligned}\Phi_i^2(L) = 4\Bigg[&\Big(\cosh(\beta_i L)\sin(\beta_i L)-\cos(\beta_i L)\sinh(\beta_i L)\Big)^2\\ & -2\beta_i^3 Jm^* L\Big(1-\cos(\beta_i L)\cosh(\beta_i L)\Big)\\ & \times\Big(\cosh(\beta_i L)\sin(\beta_i L)-\cos(\beta_i L)\sinh(\beta_i L)\Big)\\ & +\beta_i^6 J^2 m^{*2} L^2\Big(1-\cos(\beta_i L)\cosh(\beta_i L)\Big)^2\Bigg]\,.\end{aligned} \tag{59}$$

Note that this form of $\Phi_i^2(L)$ appears in Equation 57, allowing us to reduce it to

$$\begin{aligned}\int_0^L \Phi_i^2 \mathrm{d}x =& L\Big(\sin(\beta_i L)+\sinh(\beta_i L)\Big)^2 - \frac{3Lm^*}{4}\Phi_i^2(L)\\ & -\beta_i^2 Jm^* L\Big[\sin^2(\beta_i L)\sinh^2(\beta_i L)\\ & -2\beta_i L\Big(\cos(\beta_i L)-\cosh(\beta_i L)\Big)\Big(\sin(\beta_i L)+\sinh(\beta_i L)\Big)\Big]\\ & +\beta_i^6 J^2 m^{*2} L^3\Big((\cos(\beta_i L)-\cosh(\beta_i L)\Big)^2 . \end{aligned} \tag{60}$$

Combining Equations 58, 59, and 60 after some manipulation gives

$$\begin{aligned}\Lambda_i =& \frac{\beta_i^4 Lm^*}{4}\Phi_i^2(L)+\beta_i^4 L\Big(\sin(\beta_i L)+\sinh(\beta_i L)\Big)^2\\ & -\beta_i^6 Jm^* L\Big[-3\sin^2(\beta_i L)\sinh^2(\beta_i L)\\ & -2\beta_i L\Big(\cos(\beta_i L)-\cosh(\beta_i L)\Big)\Big(\sin(\beta_i L)+\sinh(\beta_i L)\Big)\Big]\\ & +\beta_i^{10} J^2 m^{*2} L^3\Big((\cos(\beta_i L)-\cosh(\beta_i L)\Big)^2 . \end{aligned} \tag{61}$$

Which for simplicity, can be written as

$$\Lambda_i = \frac{\beta_i^4 Lm^*}{4}\Phi_i^2(L)+\beta_i^4 L\Big(\sin(\beta_i L)+\sinh(\beta_i L)\Big)^2+\beta_i^6 Jm^* Lf(m^*,J)\,, \tag{62}$$

where

$$\begin{aligned}f(m^*,J) =& 3\sin^2(\beta_i L)\sinh^2(\beta_i L)\\ & +2\beta_i L\Big(\cos(\beta_i L)-\cosh(\beta_i L)\Big)\Big(\sin(\beta_i L)+\sinh(\beta_i L)\Big)\\ & +\beta_i^4 Jm^* L^2\Big((\cos(\beta_i L)-\cosh(\beta_i L)\Big)^2 . \end{aligned} \tag{63}$$

By substituting Equation 42 into Equation 59 and rearranging we get

$$
\begin{aligned}
\Phi_i^2(L) =& 4\Big(\sin(\beta_i L)+\sinh(\beta_i L)\Big)^2 \\
&- 8\beta_i L m^* \sin(\beta_i L)\sinh(\beta_i L) \\
&\times \Big(\cosh(\beta_i L)\sin(\beta_i L)-\cos(\beta_i L)\sinh(\beta_i L)\Big) \\
&+ 8\beta_i^3 L J m^* \Big[-\Big(1-\cos(\beta_i L)\cosh(\beta_i L)\Big) \\
&\times \Big(\cosh(\beta_i L)\sin(\beta_i L)-\cos(\beta_i L)\sinh(\beta_i L)\Big) \\
&- \sin(\beta_i L)\sinh(\beta_i L)\Big(\cosh(\beta_i L)\sin(\beta_i L)+\cos(\beta_i L)\sinh(\beta_i L)\Big) \\
&+ \beta_i L m^* \Big(1-\cos(\beta_i L)\cosh(\beta_i L)\Big)\sin(\beta_i L)\sinh(\beta_i L)\Big] \\
&+ 4\beta_i^6 L^2 J^2 m^{*2}\Big(1-\cos(\beta_i L)\cosh(\beta_i L)\Big)^2 .
\end{aligned}
\tag{64}
$$

Initially this form appears to be more complicated. However, as will be demonstrated, because it contains the boundary conditions it produces a final result that is more physically understandable. For simplicity it can be written as

$$
=4\Big[\Big(\sin(\beta_i L)+\sinh(\beta_i L)\Big)^2-\beta_i L m^*\Big(2g+\beta_i^2 J h(m^*,J)\Big)\Big], \tag{65}
$$

where

$$
g=\sin(\beta_i L)\sinh(\beta_i L)\Big(\cosh(\beta_i L)\sin(\beta_i L)-\cos(\beta_i L)\sinh(\beta_i L)\Big) \tag{66}
$$

and

$$h(m^*,J) = 2\Big[-\Big(1-\cos(\beta_i L)\cosh(\beta_i L)\Big) \\ \times\Big(\cosh(\beta_i L)\sin(\beta_i L)-\cos(\beta_i L)\sinh(\beta_i L)\Big) \\ -\sin(\beta_i L)\sinh(\beta_i L)\Big(\cosh(\beta_i L)\sin(\beta_i L)+\cos(\beta_i L)\sinh(\beta_i L)\Big) \\ +\beta_i L m^*\Big(1-\cos(\beta_i L)\cosh(\beta_i L)\Big)\sin(\beta_i L)\sinh(\beta_i L)\Big] \\ +\beta_i^3 L J m^*\Big(1-\cos(\beta_i L)\cosh(\beta_i L)\Big)^2 \tag{67}$$

Thus, finally we can write

$$\frac{\Lambda_i L^3}{\Phi_i^2(L)} = \frac{\beta_i^4 L^4}{4}\left(m^* + \frac{\Big(\sin(\beta_i L)+\sinh(\beta_i L)\Big)^2+\beta_i^2 J m^* f(m^*,J)}{\Big(\sin(\beta_i L)+\sinh(\beta_i L)\Big)^2-\beta_i L m^*\Big(2g+\beta_i^2 J h(m^*,J)\Big)}\right). \tag{68}$$

It is clear that in the case of no tip, $m^* = 0$, this reduces to just $\frac{\beta_i^4 L^4}{4}$. Inserting this into Equation 16 yields a result consistent with Melcher et al. [2]. Further consistency with the literature can be shown in the case of the point mass, where $J = 0$ but $m^* \neq 0$. Inserting Equation 68 into Equation 16 with these conditions, agrees with the results of Lozano et al. [3].

A final test of the accuracy of this equation can be done by considering equipartition theorem with Hooke's law in terms of the static spring constant:

$$\frac{1}{2}k_B T = \frac{1}{2}k_{\text{stat}}\sum_{i=1}^{\infty}\left\langle Z_i^2(L)\right\rangle \tag{69}$$

and inserting Equation 15 we get

$$\sum_{i=1}^{\infty}\frac{L^3\Lambda_i}{\Phi_i^2(L)} = 3\,. \tag{70}$$

Using the same model qPlus sensor as described above, this has been plotted in Figure 1, for $i =$ 1..8 showing excellent agreement with theory, and faster convergence for larger tips.

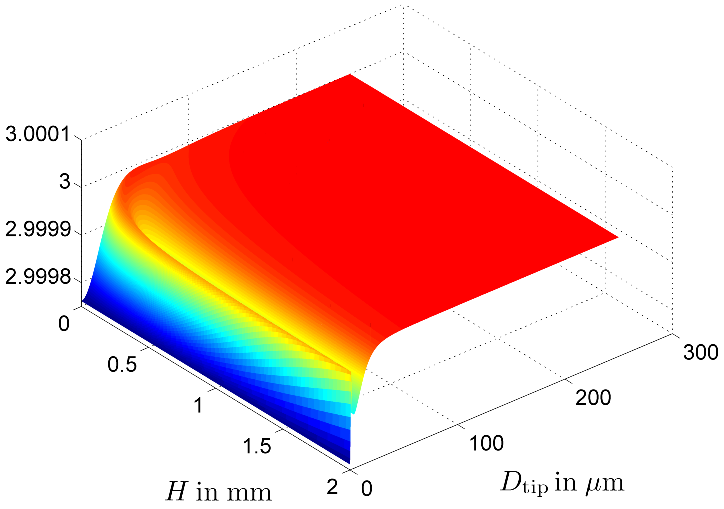

Figure S 1: Agreement with $\lim_{N\to\infty} \sum_{i=1}^{N} \frac{L^3 \Lambda_i}{\Phi_i^2(L)} = 3$, plotted for $N = 8$ roots to show that the equation is consistent with equipartition theorem. Where H is the length and D_{tip} is the diameter of a cylindrical tip.

References

1. Butt, H. J.; Jaschke, M. *Nanotechnology* **1995**, *6*, 1. doi:10.1088/0957-4484/6/1/001.

2. Melcher, J.; Hu, S.; Raman, A. *Appl. Phys. Lett.* **2007**, *91*, 053101. doi:10.1063/1.2767173.

3. Lozano, J. R.; Kiracofe, D.; Melcher, J.; Garcia, R.; Raman, A. *Nanotechnology* **2010**, *21*, 465502. doi:10.1088/0957-4484/21/46/465502.
